# Methylation of Imprinted Genes in Sperm DNA Correlated to Urinary Polycyclic Aromatic Hydrocarbons (PAHs) Exposure Levels in Reproductive-Aged Men and the Birth Outcomes of the Offspring

**DOI:** 10.3389/fgene.2020.611276

**Published:** 2021-01-11

**Authors:** Jia Yang, Zhaoxu Lu, Zhichao Liu, Li Wang, Mei Qiang

**Affiliations:** Department of Children and Adolescences Health, School of Public Health, Shanxi Medical University, Taiyuan, China

**Keywords:** polycyclic aromatic hydrocarbons, birth outcomes, imprinted genes, methylation, sperm

## Abstract

Polycyclic aromatic hydrocarbons (PAHs) are known environmental pollutants. Studies are very limited regarding the impacts of paternal PAHs exposure on birth outcomes as well as the underpinning mechanisms in human. In this study, 302 reproductive-aged males (22–46 years old) were enrolled and demographic informatics data were obtained by questionnaires. The levels of urinary hydroxylated PAHs (OH-PAHs) were assessed by ultra-high performance liquid chromatography-tandem mass spectrometry; and methylation levels of the imprinting genes *H19*, *Meg3*, and *Peg3* of sperm DNA were evaluated via bisulfite pyrosequencing. The analysis of the correlation between OH-PAHs levels and methylation levels of imprinting genes showed that OH-PAHs are correlated with some CpG sites in *H19, Peg3*, and *Meg3*. To further investigate an association of urinary OH-PAHs with birth outcomes, follow-up study of wives of these subjects has been performed for 1–3 years. As the result, a total of 157 babies were born. The birth outcomes parameters including birth weight (BW), length (BL), and ponderal index (PI) were recorded. The further analysis of generalized estimating equation indicated a negative correlation between urinary total OH-PAHs levels and newborn BW (β = −0.081, *p* = 0.020); but this association has not been found for BL and PI. Furthermore, a logistic regression analysis was employed for examining associations of the methylation of imprinting genes with birth outcomes parameters, which indicated a negative correlation between BW and *H19*, namely, each unit percent (%) elevation in methylation of *H19* (but not *Peg3* and *Meg3*) was significantly associated with a 0.135 g reduction of BW (β = −0.135; 95% CI 0.781–0.978). Putting together, these results show that paternal non-occupational environmental exposure to PAHs is associated with newborn BW. And imprinting gene *H19* methylation may be involved in the underlying mechanisms. This study in human population adds a support for previous animal study and implies that environmental impact on the offspring through paternal pathway.

## Introduction

Recently, the impacts of environmental pollutants on reproduction and development in humans have been of increasing concern (Nassar et al., 2010; Shirangi et al., 2011). Polycyclic aromatic hydrocarbons (PAHs) represent molecules released during partial combustion of hydrocarbons (Mumtaz and George, 1995). Outdoor air and soil could contain PAHs produced by industries, wild fires, automobiles, and asphalt, while indoor air could also be polluted with PAHs by home heating and cooking emissions (Lewtas, 2007). Human exposure to PAHs occurs through air inhalation, ingested food (especially after meat grilling or smoking), and exposure to tobacco smoke (US Department of Health Human Services, 1995; Aquilina et al., 2010). PAHs are metabolically bio-transformed in the body to produce hydroxylated compounds that ultimately undergo urine excretion. In human study, urine contents of hydroxylated PAHs (OH-PAHs) have been broadly employed as surrogates for estimating exposure to PAHs (Ramesh et al., 2004). Since PAHs can cross the placental barrier into the fetus, these compounds have also been proven to lead detrimental effects such as congenital malformation and developmental disorders (Polanska et al., 2014; Rengarajan et al., 2015). For example, previous animal studies suggested that PAHs affect the length of gestation and intrauterine growth (Detmar et al., 2008). Further human studies showed direct maternal exposure to PAHs of pregnant women was associated with adverse birth outcomes (Dejmek et al., 2000; Choi et al., 2006, 2008; Lamichhane et al., 2016), including delayed intrauterine growth and preterm deliveries (Singh et al., 2008; Wilhelm et al., 2011; Guo et al., 2012), reduced newborn weights, head circumferences, and lengths (Dejmek et al., 2000; Perera et al., 2005; Choi et al., 2006; Tang et al., 2006; Jedrychowski et al., 2012, 2017; Duarte-Salles et al., 2013; Langlois et al., 2014; Padula et al., 2014). However, research on adverse birth outcomes from paternal PAHs exposure is very limited (Padula et al., 2014). Recently, paternal impacts on embryonic development is receiving increased attention. The mechanisms underlying the impact of paternal PAHs exposure on their offspring have not been fully characterized.

Imprinted genes play critical roles in normal fetal growth and placental function (Tunster et al., 2013). It has been suggested that epigenetic events might underlie the risk of adverse birth outcomes through phenotypic plasticity (Vidal et al., 2014; Montoya-Williams et al., 2017). A previous study revealed that paternal life behavior and the effects of environmental exposure are passed on to future generations through sperm genetic imprinting (Abbasi, 2017). The large number of imprinted genes known to affect fetal and placental growth. Among them, research reports showed that disturbance of the methylation status of the imprinted genes *H19* and *Mest* leads to decreased sperm function and embryo development failure, as well as with the risk of passing this defect on to the offspring (Marques et al., 2004). Paternally expressed genes, such as *Peg3*, is susceptible to epigenetic modification, are highly expressed in the placenta, which is critical for fetal growth (Joomyeong et al., 2013). In additional, previous study showed that a small group of imprinted genes including *Meg3* were affected in IUGR placentas (McMinn et al., 2006). Therefore, the current study will address epigenetical mechanisms underlying the associations of paternal urine PAHs contents with adverse birth outcomes by examining the relevant changes of the imprinting genes *H19*, *Peg3*, and *Meg3* in human sperm DNA.

Our previous animal experimental studies of paternal B(a)P exposure showed a disorder in the methylation levels of four imprinting genes in sperm DNA. Importantly, a similar pattern in methylation change of these genes was seen in their unexposed F1-2 male mice (Zhang et al., 2019). In our recent human studies, paternal Hg and PAHs exposures are associated with altered methylation pattern in sperm (Lu et al., 2018; Ma et al., 2019). However, whether paternal exposure to PAHs and the altered DNA methylation levels of imprinting genes in sperm are linked, and eventually lead to adverse birth outcomes, remains unknown. Shanxi Province of China represents an industrialized region with relatively elevated amounts of PAHs in air, especially due to coal-processing waste. By investigating the father and their newborns in this area, current study aimed to assess potential associations of paternal PAHs exposure with birth outcomes.

## Materials and Methods

### Study Participants and Data Collection

A total of 302 reproductive-age men were enrolled at the Reproductive Clinic of Maternal and Child Care Service Center in Shanxi Province from April 2015 to March 2016. They visited the center for pre-pregnancy check or family infertility exam. After enrollment, the participants completed a validated questionnaire including sociodemographic information (age, occupation, education level, history of abnormal pregnancy, and health condition) and lifestyles (smoking and consumption of alcohol, and frequencies of consuming fried and barbecued meats). The exclusion criterion was a family history of birth defects. The included individuals were followed up until their children were born, and newborn conditions (birth outcome, vital signs, and delivery conditions). All participating individuals provided written informed consent. The study protocol had approval from the Research Ethics Committee of Shanxi Medical University.

### Urine and Semen Collection and Analysis

#### Specimen Collection

A total of 50 ml midstream urine was obtained in clean vessels followed by immediate transport to the laboratory, and repackaging into and storage in 10 mL vials at −80°C. Meanwhile, semen specimens were collected after refraining from ejaculation for 3–7 days, by masturbation with no lubricant use into sterile tubes. Upon liquefaction, semen specimens underwent centrifugation (200 g, 15 min), and the pelleted sperm was kept at −80°C for subsequent DNA methylation measurement.

#### Assessment of Urinary Monohydroxylated PAHs Levels

The PAHs metabolites measured in the present study included 1-hydroxynaphthalene (1-OH NAP), 2-hydroxynaphthalene (2-OH NAP), 3-hydroxyfluoren (3-OH FLU), 2-hydroxyfluoren (2-OH FLU), 2-hydroxyphenanthrene (2-OH PHE), 1-hydroxyphenanthrene (1-OH PHE), 1-hydroxypyrene (1-OH PYR). For assessment, enzyme digestion and solid phase extraction were carried out, followed by Ultra-high Performance Liquid Chromatography with tandem Mass Spectrometry (UHPLC-MS/MS; Shimadzu, Japan). For enzymatic hydrolysis, 4 mL of thawed urine sample was mixed with 4 mL of acetic acid-ammonium acetate buffer solution (pH = 5) and 20 μL of β-glucuronidase (Sigma, United States), and then incubated at 37°C for 12 h. Then, the mixture was loaded onto a Sep-Pak C18 (6 cc, 500 mg) cartridge (Waters, United States), which was activated with 5 mL of methanol and 5 mL of distilled water. The hydrolyzate was enriched using a C18 solid-phase extraction column eluted with 5 mL of 30% methanol, and slowly eluted with 5 mL of methanol. The eluate was concentrated by dry nitrogen purge and fixed by methanol to yield the extract (0.5 ml). UHPLC-MS/MS was performed to simultaneously measure the seven OH-PAHs as described previously (Zhao et al., 2013; Lankova et al., 2016; Urbancova et al., 2017). The limit of detection (LOD) was the lowest standard concentration yielding a signal-to-noise ratio (S/N) of three. Linearity (R2), LOD, precision (relative standard deviation or RSD), and mean recovery rate were 0.9930–0.9998, 0.02–0.094 ng/mL, 2.7–11.6%, and 71.4–109.4%, respectively. Reagent blanks and urine specimens were assessed, and none of the PAH metabolites were found, suggesting the absence of contamination.

#### DNA Extraction and Bisulfite Treatment

Genomic DNA from each individual sperm sample was extracted with QIAamp DNA micro kit (Qiagen, United States) as previously proposed (Yuan et al., 2015). DNA quantitation was carried out on a Nanodrop 2000 Spectrophotometer (Thermo Fisher Scientific). Then, genomic DNA (1,000 ng) underwent treatment with sodium bisulfite for converting unmethylated cytosine moieties into uracil while leaving methylated cytosine unaltered, with EZ Methylation Gold-Kit (Zymo Research, United States) as directed by the manufacturer. After the converted reaction, DNA elution was performed with 15 μl of TE buffer (10 mM Tris–HCl, 0.1 mM EDTA, pH 7.5), and employed for PCR amplification.

#### PCR Amplification of Bisulfite-Treated Sperm DNA and Pyrosequencing

The experimental protocols were described in a previous report (Lu et al., 2018). Briefly, PCR was performed with Pyro Mark PCR Kit (Qiagen, CA, United States) with about 50 ng of bisulfite-DNA and 0.2 μM each of forward and reverse primers in 25 μl PCR reactions, as directed by the manufacturer. Reverse primers were linked to biotin; pyrosequencing was carried out on a Pyro Mark Q96 MD pyrosequencing instrument (Qiagen). PCR and pyrosequencing primers are shown in Table 1.

**TABLE 1:** Primer sequences.

DMR (Chr)	Forward primers	Reverse primers*	Sequencing primers
*H19* (11p15.5)	GTATATGGGTATTTTTTGGAGGT	ATATCCTATTCCCAAATAA	TGGTTGTAGTTGTGGAAT
*Meg3* (14q32.3)	GGGATTTTTGTTTTTTTTTGTAGTAGG	CCAACCAAAACCCACCTATAAC	TTTGGGGTTGGGGTT
*Peg3* (19q13.43)	TAATGAAAGTGTTTGAGATTTGTTG	CCTATAAACAACCCCACACCTATAC	GGGGGTAGTTGAGGTT

*Biotin tagged primer.

The biotinylated amplicons were obtained with streptavidin-Sepharose beads (Amersham, United States) and underwent sequencing with PyroMark Gold Q96 kit (Biotage, Sweden). Methylation levels at various CpG sites were examined with the PyroMark CpG Software (Biotage). Duplicate pyrosequencing assays were carried out in sequential runs. The numbers of CpGs sites assessed at various differentially methylated regions (DMRs) were: *H19*, 7; *Meg3*, 8; *Peg3*, 8 (Edwards et al., 2006).

### Anthropometric Assessment of Newborns

Birth outcomes from follow-up investigation were assessed. The anthropometric parameters of newborns were birth weight (BW), birth length (BL), and ponderal index (PI). The PI {BW (g)/[BL (cm)] 3 × 102} is a body mass index that is most commonly used in pediatrics (Leitner et al., 2007).

### Statistical Analysis

Hydroxylated PAHs levels were presented as median and interquartile range (IQR). The concentrations of PAHs metabolites below the LOD were considered to be half the indicated LOD. The Pearson correlation method was performed for evaluating associations of methylation at various CpG sites. Generalized estimating equation (GEE) analysis was performed for determining associations of urine OH-PAHs levels with the offspring’s birth outcomes after adjusting for potential confounding factors. The associations of paternal OH-PAHs levels with the methylation levels of the imprinting genes *H19*, *Meg3*, and *Peg3* in sperm DNA were examined with Logistic regression analysis with the above co-variates to rule out potential confounders of BW after controlling for potential confounders using a logistic regression model. SPSS for Windows 22.0 (SPSS, United States) was employed for statistical analyses. Bilateral *p* < 0.05 indicated statistical significance.

## Results

### Demographic Features of the Participants

A total of 302 male participants in reproductive-age from 22 to 46 years old (median age of 30 years) were recruited. Their baseline features are presented in Table 2. Of all participant individuals, 54.0% had a college education level or higher; there were 30.1% with drinking status, and 55.7% were overweight (BMI > 24). More than half of the participants (167) were smokers, with 158 having three or more years of smoking experience; only 10.9% (*n* = 33) ate smoked food.

**TABLE 2:** Profiles of the 302 male participants in this study.

Variables	Mean ± SD or *N* (%)
Age (years)	30.4 ± 4.1
BMI (kg/m^2^)	24.9 ± 4.2
<18.5	14 (4.6)
18.5–23.9	120 (39.7)
24–27.9	99 (32.8)
28–32	57 (18.9)
>32	12 (4.0)
**Education**	
Primary school	4 (1.3)
Middle school	69 (24.1)
High school graduate	66 (21.9)
College or above	163 (54.0)
Smoking	167 (55.3)
**Smoking years**	
0–3	144 (47.7)
>3	158 (52.3)
Drinking	91 (30.1)
Eating smoked food	33 (10.9)
Drinking coffee	23 (7.6)
Drinking tea	134 (44.4)

### Concentration Distributions of the Seven OH-PAHs in Urine

To examine PAH exposure level, seven OH-PAHs were measured in urinary samples from the 302 participants. The concentrations corrected by urine specific gravity (Leitner et al., 2007; Gluckman et al., 2009; El Hajj et al., 2014) are shown in Table 3. The medians and IQRs of these OH-PAHs were as follows: 2-OH NAP, 0.058 (0.020–0.142); 1-OH NAP, 0.027 (0.009–0.049); 3-OH FLU, 0.025 (0.015–0.044); 2-OH FLU, 0.032 (0.019–0.062); 2-OH PHE, 0.092 (0.044–0.187); 1-OH PHE, 0.006 (0.001–0.027); 1-OH PYR, 0.015 (0.002–0.049). Of these OH-PAHs, the median value of 2-OH PHE was the highest (0.092 μg/L), then followed by urinary 2-OH NAP (0.058 μg/L), and urinary 2-OH FLU (0.032 μg/L). While the median concentration of urinary 1-OH PYR was the lowest (0.015 μg/L).

**TABLE 3:** The concentrations of seven OH-PAHs in the urine sample (μg/L).

	*N*	P25	Median	P75	Range
2-OH NAP	302	0.020	0.058	0.142	0.001–130.485
1-OH NAP	302	0.009	0.027	0.049	0.000–2.888
3-OH FLU	302	0.015	0.025	0.044	0.000–0.733
2-OH FLU	302	0.019	0.032	0.062	0.000–0.840
2-OH PHE	302	0.044	0.092	0.187	0.002–2.146
1-OH PHE	302	0.001	0.006	0.027	0.000–0.365
1-OH PHY	302	0.002	0.015	0.049	0.000–1.674
ΣOH PAH^1^	302	0.199	0.348	0.713	0.030–133.67

*^1^Sum of the seven hydroxy-metabolites of PAHs.*

### Methylation Levels of Imprinting Genes in Sperm DNA

For the 302 participants, DNA methylation levels of *H19*, *Peg3*, and *Meg3* measured in sperm samples are presented as mean ± standard deviation (Table 4). The average methylation levels of all analyzed CpG sites in *H19, Peg3*, and *Meg3* were 87.55, 0.99, and 2.25%, respectively. To further determine potential correlations among individual CpG sites and between individual CpG sites and their average methylation levels for *H19*, *Peg3*, and *Meg3*, respectively, we performed the Pearson correlation analyses among seven CpG sites in *H19*, seven CpG sites in *Peg3* and eight CpG sites in *Meg3*. DNA methylation levels in more than half of CpG sites within the *H19* gene and all sites in *Peg3* and *Meg3* were positively correlated (*r* = 0.882–0.986, *p* < 0.05–0.01) (Table 5). Additionally, average methylation levels were also strongly correlated with relevant CpG sites (*r* = 0.217–0.994, *p* < 0.05–0.01). Thus, average methylation levels were employed to further assess associations.

**TABLE 4:** Methylation levels in the sperm DNA (%).

*N*		Mean ± SD								
		CpG1	CpG2	CpG3	CpG4	CpG5	CpG6	CpG7	CpG8	Average of analyzed CpG sites
*H19*	301^*a*^	89.26 ± 5.57	97.64 ± 3.17	72.60 ± 8.13	69.13 ± 26.87	90.69 ± 6.03	96.91 ± 9.99	97.20 ± 8.65		87.55 ± 4.63
*Peg3*	302	1.78 ± 3.42	0.90 ± 2.89	1.03 ± 3.17	1.18 ± 3.49	0.69 ± 2.73	0.74 ± 2.85	0.63 ± 2.86		0.99 ± 2.96
*Meg3*	302	1.29 ± 3.74	4.10 ± 4.65	2.24 ± 5.28	3.16 ± 4.84	1.44 ± 4.97	1.69 ± 5.29	2.20 ± 5.30	1.86 ± 5.26	2.25 ± 4.84

*^*a*^One sample was excluded for *H19* methylation in sperm. The sample was affected by technical errors according to quality assessment by the PyroMark Q96 software.*

**TABLE 5:** Pearson correlation coefficients of sperm DNA methylation at the CpG sites.

	*H19* (*N* = 301)								*Peg3* (*N* = 302)								Meg*3* (*N* = 302)								
	CpG1	CpG2	CpG3	CpG4	CpG 5	CpG6	CpG7	Average	CpG1	CpG2	CpG3	CpG4	CpG5	CpG6	CpG7	Average	CpG1	CpG2	CpG3	CpG4	CpG5	CpG6	CpG7	CpG8	Average
CpG1	1	0.215**	0.424**	0.021	0.410**	–0.010	–0.086	0.382**	1	0.916**	0.930**	0.917**	0.893**	0.911**	0.882**	0.955**	1	0.935**	0.980**	0.970**	0.968**	0.985**	0.971**	0.975**	0.987**
CpG2	–	1	–0.022	–0.003	–0.038	0.102	0.206**	0.217**	–	1	0.942**	0.947**	0.935**	0.935**	0.923**	0.974**	–	1	0.929**	0.948**	0.933**	0.939**	0.928**	0.933**	0.957**
CpG3	–	–	1	−0.490**	0.288**	0.007	0.035	−0.004**	–	–	1	0.944**	0.906**	0.940**	0.922**	0.973**	–	–	1	0.974**	0.976**	0.986**	0.978**	0.977**	0.990**
CpG4	–	–	–	1	0.174**	–0.014	–0.070	0.706**	–	–	–	1	0.940**	0.945**	0.917**	0.977**	–	–	–	1	0.966**	0.975**	0.975**	0.968**	0.987**
CpG5	–	–	–	–	1	−0.113*	–0.106	0.387**	–	–	–	–	1	0.925**	0.916**	0.961**	–	–	–	–	1	0.983**	0.979**	0.980**	0.988**
CpG6	–	–	–	–	–	1	0.044	0.390**	–	–	–	–	–	1	0.920**	0.970**	–	–	–	–	–	1	0.981**	0.980**	0.994**
CpG7							1	0.228**							1	0.956**							1	0.975**	0.989**
CpG8																								1	0.989**

**p < 0.01 (double-tailed), *p < 0.05 level (double-tailed).

### Association Between Sperm DNA Methylation and OH-PAHs Concentrations

To examine potential relationship between DNA methylation levels of selected imprinting genes and urinary OH-PAHs concentrations, Spearman’s rank correlation analysis was carried out by using average methylation level of selected CpGs sites in each gene. The results indicated that there were associations between some individual CpG sites on *H19, Peg3*, and *Meg3* DMR and OH-PAHs concentrations. In *H19*, we found multiple sites correlated with OH-PAHs concentrations. Namely, there were positive correlation found at CpG #1 between in the level of methylation and 1-OH PHY (rs = 0.243, *p* = 0.002); at CpG #5 between the level of methylation and 1-OH NAP (rs = 0.157, *p* = 0.05) and 2-OH PHE (rs = 0.186, *p* = 0.02); at CpG #6 between the level of methylation and 3-OH FLU (rs = 0.188, *p* = 0.019). While a negative correlation between the level of 1-OH PHE and methylation at CpG #3 (rs = −0.302, *p* < 0.001) and CpG #5 (rs = −0.279, *p* < 0.001). In *Peg3*, methylation levels at CpG #1 (rs = 0.175, *p* = 0.029), CpG #2 (rs = 0.194, *p* = 0.015), CpG #3 (rs = 0.160, *p* = 0.046), and CpG #5 (rs = 0.214, *p* = 0.007) of demonstrated a positive correlation with the levels of 2-OH FLU, respectively. And methylation levels at CpG #2 of *Meg3* demonstrated a positive correlation with 1-OH PHE (rs = 0.301, *p* = 0.02) and 2-OHPHE (rs = 0.173, *p* = 0.03), respectively (Table 6). These results imply the associations between DNA methylation levels of selected imprinting genes and OH-PAHs concentrations.

**TABLE 6:** Spearman correlation analysis between individual methylation levels CpGs of *H19, Peg3, and Meg3 and OH-PAHs*

	*H19*							*Peg3*							*Meg3*							
	CpG1	CpG2	CpG3	CpG 4	CpG 5	CpG 6	CpG 7	CpG 1	CpG 2	CpG 3	CpG 4	CpG 5	CpG 6	CpG 7	CpG 1	CpG 2	CpG 3	CpG 4	CpG 5	CpG 6	CpG 7	CpG 8
2-OHNAP	0.026	0.010	0.112	0.011	0.105	–0.018	–0.111	0.099	0.041	0.015	0.007	0.037	0.004	0.022	0.023	–0.016	0.010	–0.029	–0.009	0.028	0.026	0.027
1-OHNAP	–0.001	0.117	–0.053	–0.023	0.157*	0.141	0.048	–0.049	–0.074	–0.107	–0.089	0.001	–0.063	0.096	–0.007	0.138	–0.122	–0.066	0.14	0.064	–0.092	–0.042
3-OHFLU	0.086	0.150	–0.105	–0.002	0.123	0.188*	0.037	–0.003	0.091	0.014	–0.054	–0.003	–0.002	0.094	0.088	0.134	–0.099	0.006	0.054	0.051	–0.032	0.005
2-OHFLU	0.047	–0.008	0.069	0.134	0.065	–0.095	–0.087	0.175*	0.194*	0.160*	0.156	0.214*	0.220	0.148	0.187	0.077	0.149	0.088	0.139	0.127	0.113	0.117
2-OHPHE	–0.113	0.006	–0.128	–0.010	0.186*	0.033	0.072	0.050	0.087	–0.016	–0.037	0.041	0.027	0.013	0.165	0.173*	0.070	0.136	0.106	0.100	0.022	0.115
1-OHPHE	–0.153	0.069	−0.302*	–0.049	−0.279*	0.120	0.154	–0.029	0.016	0.007	–0.092	–0.033	–0.027	0.017	0.134	0.301*	–0.047	0.114	0.141	0.116	–0.092	0.094
1-OHPHY	0.243*	0.060	0.037	0.036	0.080	0.071	–0.149	0.114	0.093	–0.034	0.031	0.085	–0.003	–0.007	–0.006	0.033	–0.038	–0.023	0.028	0.020	–0.078	–0.062

*p < 0.05 level (double-tailed).

### Associations of Paternal Urine OH-PAHs Concentrations With Birth Outcomes in the Newborns

These 302 reproductive-aged men were then followed up for 1–3 years to record the development of their offspring. A total of 157 out of the 302 participants’ wives were pregnant and successively gave birth. The babies were born with the mean BW of 3,169.3 g (±643.8 g) and BL of 49.8 cm (±2.9 cm). The percentage of newborn girls was slightly lower compared with that of boys (42.7 and 57.3%, respectively). Three parameters of BW (*t* = −0.477, *p* = 0.634), BL (*t* = −0.044, *p* = 0.965), and PI (*t* = −0.951, *p* = 0.343) of the newborns at delivery were comparable in girls and boys.

**FIGURE 1:**
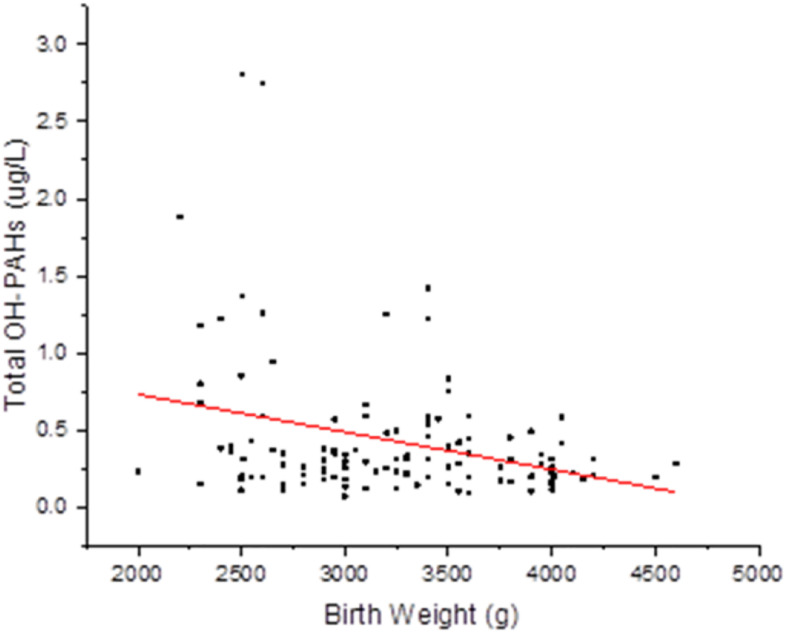
Correlation between total OH-PAHs levels and new born BW. Total OH-PAHs levels were negative correlated to new born BW in this study. Each point on the graph represents individual measurement and red line represents Liner fit trend.

To evaluate potential associations of paternal urinary OH-PAHs levels with offspring’s birth outcomes parameters, we divided the newborns into two groups based on BW: Group 1, 2,500–4,000 g, and Group 2, <2,500 g or >4,000 g. Group 1 had 129 individuals and Group 2 had 28. In addition, we divided the newborns into two groups by BL: Group 1, 45.2–55.8 cm, and Group 2, <45.2 cm or >55.8 cm. Also, based on PI, the newborns were divided into: Group 1, 2.0–2.2, and Group 2, <2.0 or >2.2. With GEE regression analysis, we found that the total OH-PAHs concentration was negatively correlated with newborns’ BW (β = −0.081, *p* = 0.020) (Supplementary Table 1 and Figure 1) after adjusting for paternal education level, paternal BMI, paternal age, paternal status of smoking (yes or no), paternal status of drinking (yes or no), paternal status of eating bacon (yes or no), newborn gender, length of gestational weeks, and delivery method. Using Multivariate procedures in the above described co-variables to control potential confounding. However, no association between the total OH-PAHs concentration and BL and PI (Supplementary Tables 2, 3) was observed.

### Associations of Sperm DNA Methylation With Newborn Birth Weight

**FIGURE 2:**
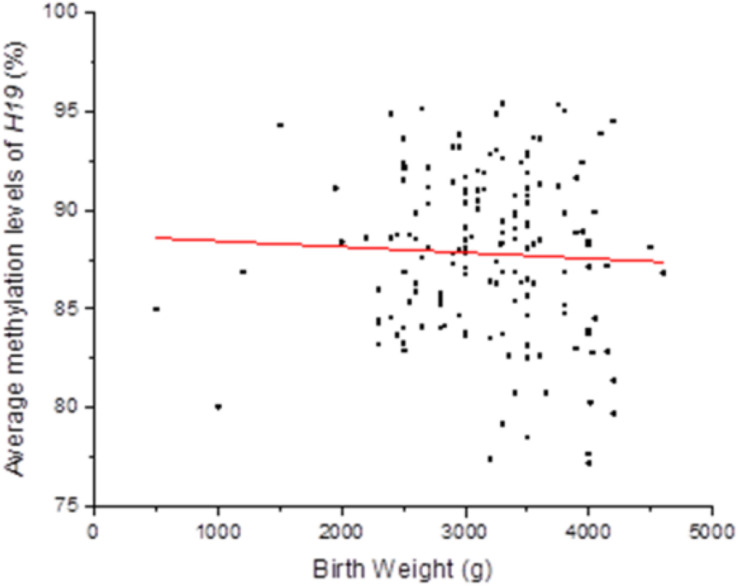
Correlation between methylation levels of imprinting gene *H19* in Sperm DNA and new born BW. Methylation levels of imprinting gene *H19* in Sperm DNA were negative correlated to new born BW in this study. Each point on the graph represents individual measurement and red line represents Liner fit trend.

Persistent epigenetic changes are considered a mechanism underlying the effects of environmental risk factors on infants (Gluckman et al., 2009; El Hajj et al., 2014). Since methylation is subjected to many factors, co-variates, such as paternal age, status of smoking, status of drinking, education level, BMI, newborn gender, gestational weeks, and delivery method, the interactions of total OH-PAHs concentration with average methylation levels of *H19*, *Peg3*, and *Meg3* and were evaluated, respectively, to understand potential mechanism underlying the associations between OH-PAHs concentration and BW. Logistic regression analysis was carried out with the above co-variates to rule out potential confounders of BW. Potential confounding factors (paternal age, paternal status of smoking, paternal status of drinking, paternal education, paternal BMI, newborn gender, gestational weeks, delivery method, interaction of total PAHs concentration with mean of *H19* methylation levels, interaction of total PAHs concentration with mean of *Peg3* methylation levels, and interaction of total PAHs concentration with mean *Meg3* methylation level) were included in the models. We found a significant negative correlation between mean *H19* methylation and BW (β = −0.135, *p* = 0.019). Namely each unit percent (%) elevation in *H19* methylation was significantly associated with 0.135 g reduction of BW (β = −0.135; 95% CI 0.781–0.978) (Table 7 and Figure 2). However, such an association was not observed for *Meg3* or *Peg3* in this study.

**TABLE 7:** Logistic regression analysis of sperm DNA methylation and newborn BW.

Factors	β	SE	Wald χ^2^	*p*	OR (95% CI)
Gestation	1.045	0.255	16.760	0.000	
37–42 weeks					1
>42 weeks					
28–37 weeks					2.844 (1.742, 4.690)
Gender	−1.106	0.541	4.177	0.041	
Male					1
Female					0.331 (0.115, 0.956)
*H19*-M	−0.135	0.057	5.532	0.019	0.874 (0.781, 0.978)

## Discussion

Recently, there has been emerging evidence that the profound paternal adverse exposure, such as obesity (Ost et al., 2014), ethanol exposure (Knezovich and Ramsay, 2012) lead an influence on the fetus growth. The results from our previous animal study indicated that paternal exposure to BaP, an important component of PAHs, induced changes in methylation levels of the four imprinting genes in sperm DNA and these alterations pasted to their F1 and F2 male offspring (Zhang et al., 2019). In the present study, we sought to provide evidence in human population by studying the impact of paternal natural exposure to environmental insult, PAHs on birth outcomes and address its underlying epigenetic mechanism. We found that a positive correlation between paternal urinary OH-PAHs level and some CpG sites in *H19, Peg3*, and *Meg3*. By following up 1–3 years, for the first time, we showed that both paternal urinary OH-PAHs and methylation levels of imprinting gene *H19* negatively correlation with body weight of newborns. These results support that paternal-PAHs exposure impacts on the fetus growth, which may be mediated by potential epigenetic mechanism.

Environmental PAHs exposure induces alterations in imprinting genes in sperm DNA.

Direct environmental exposures have different impacts on exposed individuals in terms of somatic cell development. However, only changes in the germ line (sperm or egg) may explain the transgenerational effects of paternal experiences on offspring development and fitness. Increasing amounts of evidence are showing that environmental exposure to harmful agents including PAHs has adverse effects on reproduction and development in animal and human fetuses’ studies (Vidal et al., 2014; Montoya-Williams et al., 2017). For example, epidemiological studies provided evidence that PAHs greatly affect multiple parameters of fetal development, including BW (Jedrychowski et al., 2017). Low BW results in elevated mortality and morbidity from the neonatal period to adult age, making it a main deleterious perinatal outcome (Risnes et al., 2011). Although environmental pollutants affect fetal development, the underpinning mechanisms remain undefined (Pedersen et al., 2013). In this study, paternal imprinting genes *H19* and *Meg3*, and the maternal imprinted gene *Peg3* were selected to investigate their sensitivity to PAHs exposure. By analysis of urine and semen samples, we found that a certain type of PAHs showed a correlation with some CpG sites of imprinting gene *H19, Peg3*, and *Meg3*, respectively. For instance, the methylation levels of *H19* showed at sites CpG#1, CpG #5, and #6 have positive correlations with 1-OH PHY, 1-OH NAP, 2-OH PHE, and 3-OH FLU, suggesting that the higher PAHs exposure may have the higher DNA methylation level. On the other hand, a negative correlation between the level of 1-OH PHE and methylation at CpG #3. So far, there is no direct evidence to explain which CpG sites should response for the effect of a specific parameter of fetal growth. Therefore, the present results demonstrate environmental PAHs exposure induces alterations in imprinting genes in sperm DNA. Therefore, they have potential to be biomarkers for the risk assessment of environmental exposure-induced embryonic developmental disorders. Paternal epigenetic mechanisms may mediate PAHs effects on birth outcomes in their offspring.

In mammalians, imprinting genes play roles in growth-related functions. Genomic imprinting represents an epigenetic event preferentially silencing a copy of an autosomal gene, allowing the expression of the other copy (Hutter et al., 2010). Epigenetic regulatory heavily contribute to imprinted genes expression, such as DNA methylation in DMRs (Iglesias-Platas et al., 2014). Meanwhile, DNA methylation is the most mature research mechanism among epigenetic markers of development, and highly vulnerable to environmental exposures. Recently, it was proposed that dynamic imprinted genes might have high susceptibility to environmental changes, and play an essential role in regulating plastic development and controlling cell functions via epigenetic modifications (Radford et al., 2011). In this study, we analyzed the association between paternal methylation level of the three imprinting genes and offspring BW; and between the level of paternal urinary OH-PAHs and offspring BW. The results indicated that only methylation of *H19* in sperm DNA was significantly negatively correlated with offspring BW, although some CpG sites in *Peg3* and *Meg3* was also observed to correlate with the level of paternal urinary OH-PAHs. This result supports that imprinting gene regulation exists tissue-specific (Lee, 2001) and implies *H19* DNA’s DMR as a potential biomarker of paternal environmental exposure to PAHs on fetal development and BW. Although sperm DNA methylation in *H19* correlated with offspring weight in this work, the timing and levels of such methylation alterations are still undefined. The detailed mechanism in regulating fetal growth need to be revealed in the further study.

Overall, the results implicated that sperm DNA responding to environmental elevated PAHs exposure lead an increase in methylation level of *H19* sperm DNA; which may mediate the occurrence of lower BW. Therefore, methylation alteration of *H19* of sperm DNA could represent a “signature” for the environmental insults.

### Limitation of the Present Study

Due to the reason of viability and cost, sample size was relatively small. In addition, birth outcomes data is less too. Therefore, our analysis could not go to deep insight. Further investigation is required by using more samples and more parameters.

## Data Availability Statement

The raw data supporting the conclusions of this article will be made available by the authors, without undue reservation.

## Ethics Statement

The studies involving human participants were reviewed and approved by the Research Ethics Committee of Shanxi Medical University. The patients/participants provided their written informed consent to participate in this study.

## Author Contributions

JY, LW, and MQ: conceptualization. JY and ZXL: methodology. JY: formal analysis and writing– original draft preparation. JY and ZCL: investigation. LW and MQ: writing– review and editing. MQ: supervision. MQ and JY: funding acquisition. All authors contributed to the article and approved the submitted version.

## Conflict of Interest

The authors declare that the research was conducted in the absence of any commercial or financial relationships that could be construed as a potential conflict of interest.
