# Supplementary material for: Methylation of Imprinted Genes in Sperm DNA Correlated to Urinary Polycyclic Aromatic Hydrocarbons (PAHs) Exposure Levels in Reproductive-Aged Men and the Birth Outcomes of the Offspring
Source: Front Genet. 2021 Jan 11;11:611276. doi: 10.3389/fgene.2020.611276 (PMC7834272; doi:10.3389/fgene.2020.611276)
Supplement: Supplementary file 1 [file Table_1.DOCX]

**Supplemental Table 1. GEE analysis the associations of total OH-PAHs concentration with BW**

| Parameters *ß* | | *Wald χ^2^* | *df* | *p* |
| --- | --- | --- | --- | --- |
| Paternal education |  |  |  |  |
| Primary school | 25.994 |  |  |  |
| Middle school | -0.213 | 0.098 | 1 | 0.755 |
| High school | -1.303 | 5.245 | 1 | 0.022 |
| Status of smoking 0.252 | | 0.248 | 1 | 0.618 |
| Status of drinking 0.246 | | 0.218 | 1 | 0.641 |
| Status of eating bacon -0.141 | | 0.047 | 1 | 0.829 |
| Maternal gestational weeks 2.212 | | 16.801 | 1 | 0.000 |
| Maternal delivery mode -0.040 | | 0.005 | 1 | 0.942 |
| Gender of newborn -0.956 | | 3.551 | 1 | 0.059 |
| Paternal BMI -0.093 | | 4.541 | 1 | 0.033 |
| Paternal age 0.054 | | 0.590 | 1 | 0.442 |
| Total PAHs concentration -0.081 | | 5.435 | 1 | 0.020 |
