# Supplementary material for: Methylation of Imprinted Genes in Sperm DNA Correlated to Urinary Polycyclic Aromatic Hydrocarbons (PAHs) Exposure Levels in Reproductive-Aged Men and the Birth Outcomes of the Offspring
Source: Front Genet. 2021 Jan 11;11:611276. doi: 10.3389/fgene.2020.611276 (PMC7834272; doi:10.3389/fgene.2020.611276)
Supplement: Supplementary file 2 [file Table_2.DOCX]

**Supplemental Table 2. GEE analysis the associations of total OH-PAHs concentration with BL**

| Parameters  *ß* | | *Wald χ^2^* | *df* | *p* |
| --- | --- | --- | --- | --- |
| Paternal education |  |  |  |  |
| Primary school | 19.659 |  |  |  |
| Middle school | 0.126 | 0.016 | 1 | 0.900 |
| High school | 0.061 | 0.003 | 1 | 0.954 |
| Status of smoking 0.119 | | 0.011 | 1 | 0.917 |
| Status of drinking 1.510 | | 1.227 | 1 | 0.268 |
| Status of eating bacon 0.598 | | 0.319 | 1 | 0.572 |
| Maternal gestational weeks 5.014 | | 9.650 | 1 | 0.002 |
| Maternal delivery mode - 1.653 | | 2.830 | 1 | 0.093 |
| Gender of newborn 0.771 | | 0.803 | 1 | 0.370 |
| Paternal BMI 0.241 | | 1.902 | 1 | 0.168 |
| Paternal age -0.183 | | 2.150 | 1 | 0.143 |
| Total PAHs concentration -0.022 | | 0.052 | 1 | 0.820 |
