# Supplementary material for: Methylation of Imprinted Genes in Sperm DNA Correlated to Urinary Polycyclic Aromatic Hydrocarbons (PAHs) Exposure Levels in Reproductive-Aged Men and the Birth Outcomes of the Offspring
Source: Front Genet. 2021 Jan 11;11:611276. doi: 10.3389/fgene.2020.611276 (PMC7834272; doi:10.3389/fgene.2020.611276)
Supplement: Supplementary file 3 [file Table_3.DOCX]

**Supplemental Table 3. GEE analysis the associations of total OH-PAH concentration with PI**

| Parameter *ß* | | *Wald χ^2^* | *df* | *p* |
| --- | --- | --- | --- | --- |
| Paternal education |  |  |  |  |
| Primary school | -24.534 |  |  |  |
| Middle school | 0.183 | 0.079 | 1 | 0.779 |
| High school | 0.256 | 0.180 | 1 | 0.671 |
| Status of smoking 0.097 | | 0.036 | 1 | 0.849 |
| Status of drinking -0.509 | | 0.725 | 1 | 0.394 |
| Status of eating bacon -0.220 | | 0.143 | 1 | 0.706 |
| Maternal gestational weeks -0.661 | | 1.169 | 1 | 0.280 |
| Maternal delivery mode 0.777 | | 2.174 | 1 | 0.140 |
| Gender of newborn 0.078 | | 0.020 | 1 | 0.886 |
| Paternal BMI -0.143 | | 2.763 | 1 | 0.096 |
| Paternal age 0.043 | | 0.502 | 1 | 0.479 |
| Total PAHs concentration -0.191 | | 2.245 | 1 | 0.134 |
